# Reducing COVID-19 Health Inequities by Identifying Social Needs and Clinical Deterioration of Discharged Emergency Department Patients

**DOI:** 10.5811/westjem.2022.8.55253

**Published:** 2022-10-18

**Authors:** Eleanor Graber, Shada Rouhani, Hazar Khidir, Michael De Luca, Elizabeth Noyes, Carlos Hernandez, Joe Tulip, M. Adrian Hasdiana, Guruprasad Jambaulikar, Regan Marsh, Michael Wilson

**Affiliations:** * Brigham and Women’s Hospital, Department of Emergency Medicine, Boston, Massachusetts; † Harvard Medical School, Department of Emergency Medicine, Boston, Massachusetts; ‡ Yale National Clinician Scholars Program, New Haven, Connecticut; § George Washington University School of Medicine, Department of Emergency Medicine, Washington, DC

## Abstract

**Introduction:**

The decision to discharge a patient from the hospital with confirmed or suspected coronavirus 2019 (COVID-19) is fraught with challenges. Patients who are discharged home must be both medically stable and able to safely isolate to prevent disease spread. Socioeconomically disadvantaged patient populations in particular may lack resources to safely quarantine and are at high risk for COVID-19 morbidity.

**Methods:**

We developed a telehealth follow-up program for emergency department (ED) patients who received testing for COVID-19 from April 24–June 29, 2020 and were discharged home. Patients who were discharged with a pending COVID-19 test received follow-up calls on Days 1, 4, and 8. The objective of our program was to screen and provide referrals for health-related social needs (HRSN), conduct clinical screening for worsening symptoms, and deliver risk-reduction strategies for vulnerable individuals. We conducted retrospective chart reviews on all patients in this cohort to collect demographic information, testing results, and outcomes of clinical symptom and HRSN screening. Our primary outcome measurement was the need for clinical reassessment and referral for an unmet HRSN.

**Results:**

From April 24–June 29, 2020, we made calls to 1,468 patients tested for COVID-19 and discharged home. On Day 4, we reached 67.0% of the 1,468 patients called. Of these, 15.9% were referred to a physician’s assistant (PA) out of concern for clinical worsening and 12.4% were referred to an emergency department (ED) patient navigator for HRSNs. On Day 8, we reached 81.8% of the 122 patients called. Of these, 19.7% were referred to a PA for clinical reassessment and 14.0% were referred to an ED patient navigator for HRSNs. Our intervention reached 1,069 patients, of whom 12.6% required referral for HRSNs and 1.3% (n = 14) were referred to the ED or Respiratory Illness Clinic due to concern for worsening clinical symptoms.

**Conclusion:**

In this patient population, the demand for interventions to address social needs was as high as the need for clinical reassessment. Similar ED-based programs should be considered to help support patients’ interdependent social and health needs beyond those related to COVID-19.

## INTRODUCTION

The decision to discharge a patient from the hospital with confirmed or suspected coronavirus 2019 (COVID-19) is fraught with challenges. Patients who are well enough to merit discharge from the emergency department (ED) are still at risk of poor health outcomes from COVID-19 later in their clinical course.^1^ Particularly early in the pandemic, discharged patients had less access to traditional, outpatient follow-up systems given the closure or significantly reduced hours of some primary care clinics. Additionally, patients with suspected and confirmed COVID-19 who are well enough to be discharged home can infect at-risk family members, who face six times higher odds of infection with COVID-19 compared to non-household contacts of COVID-19.^2^,^3^,^4^

Vulnerable, historically marginalized patient populations face greater risk of experiencing subsequent clinical deterioration as well as challenges in self-isolation and social distancing.^5^,^6^,^7^ These challenges, all components of a patient’s health-related social needs (HRSN), can include cohabitation with multiple family members or friends, unstable housing, food insecurity, poor access to private transportation, limited social networks, lack of child or eldercare, and reliance on income from low-wage and low-benefit essential jobs.^8^, ^9^

As a part of the health system safety net, EDs see a higher proportion of patients with unmet HRSNs relative to other care settings.^10^,^11^,^12^ Although national and international public health agencies recommend that clinicians ensure that COVID-19 patients’ living conditions support self-isolation and that patients have access to critical resources (eg, food) when making the decision to discharge patients home, the acute care setting presents unique challenges to comprehensively assessing patients’ self-isolation needs.^13^ Emergency clinicians have limited time to conduct comprehensive social needs screening and to provide up-to-date information to patients on available community resources. Many EDs do not have existing mechanisms for conducting HRSN screening and referral. Further, needs may not be apparent at the time of the ED visit, as it can be difficult for patients to anticipate what resources will be required during an isolation period.^14^

Here we describe and evaluate a telehealth follow-up program early in the pandemic to iteratively evaluate the clinical status and HRSNs of patients who were discharged from the ED after undergoing COVID-19 testing. The goals of the program were to 1) identify patients with worsening clinical symptoms who required repeat clinical evaluation, and 2) facilitate safe self-isolation by assisting patients in meeting their HRSNs, reinforcing self-isolation instructions, and providing risk-reduction strategies for at-risk individuals.

## METHODS

### Target Population

Our quality improvement (QI) program was based in two affiliated EDs: one within a large, urban, academic hospital and the other within a neighboring community hospital. Our target population was patients who underwent reverse-transcription polymerase chain reaction (RT-PCR) testing in the ED setting for severe acute respiratory syndrome coronavirus 2 (SARS-CoV-2) and were discharged home from April 24–June 29, 2020. Of note, the intervention concluded in June 2020 as cases fell substantially during that period. At that time SARS-CoV-2 testing was available to symptomatic patients only. Asymptomatic patients were not tested in the ED unless they were admitted to the hospital. Result turnaround times during this period were 24–48 hours; thus, patients were typically discharged with their results pending. Due to concern about false negative rates, patients with symptoms of SARS-CoV-2 were instructed to self-isolate regardless of test results.

**Population Health Research Capsule**

What do we already know about this issue?

*Patient populations with health-related social needs (HRSN) may lack resources to safely isolate or quarantine and are at high risk for COVID-19 morbidity.*

What was the research question?

*Can phone screening identify and refer discharged ED patients with worsening clinical symptoms or unmet HRSNs?*

What was the major finding of the study?

*Of 1,468 patients COVID + discharged patients, 17% were referred to a physician’s assistant (PA) for clinical worsening, 13% were referred to a patient navigator, and 1.3% were referred to the ED or Respiratory Clinic for clinical worsening. The demand for interventions to address social needs was as high as the need for clinical reassessment.*

How does this improve population health?

*Screening programs based in the ED could help support patients’ interdependent social and health needs, for both COVID-19 and beyond.*

## Objective

The QI program objectives were as follows: (1) identify confirmed or suspected COVID-19 patients with worsening clinical symptoms who required further evaluation, either virtually or in person; 2) identify confirmed or suspected COVID-19 patients with unmet HRSNs that might affect their ability to isolate or quarantine and refer them to community programs and social services; and 3) deliver and reinforce self-isolation counseling and risk-reduction strategies to patients and their household contacts.

### Patient Identification

Patients were identified through a report generated by the electronic health record (EHR) system of patients who were tested for SARS-CoV-2 with RT-PCR in the ED and were discharged home. Initially all patients who were tested were called regardless of test results. On June 10, as the pandemic evolved and confidence in the sensitivity of testing grew, the program began calling only patients with confirmed COVID-19.

### Intervention

Our protocol used a brief, scripted telephone call to screen for clinical symptom progression and unmet HRSNs that might compromise safe isolation. Telephone check-ins were conducted on Days 1 and 4 after the patient’s initial presentation to the ED and were conducted by ED staff, including physician assistants (PA) and research assistants (RA). Day 8 telephone check-ins were added several weeks into the program to supplement Day 1 and Day 4 calls and were conducted May 10–June 29, 2020.

On Day 1 telephone check-ins, ED PAs called suspected COVID-19 patients discharged from the ED to notify them of the results of their COVID-19 testing, screen for worsening clinical symptoms, screen for immediate HRSNs (Table 1) and, when positive, refer them to Medicaid Accountable Care Organization (ACO) ED patient navigators for a same-day social needs assessment. Prior to the pandemic, the ED patient navigators’ role was to connect Medicaid ACO patients with outpatient healthcare clinicians and to address HRSNs during and after ED treatment. The role of this program later expanded to assist all patients with HRSNs regardless of enrollment in the ACO. When the patient navigators received a referral from the ED staff, they reached patients by phone and screened for housing stability, food security, access to medications and safety, and then connected patients to resources as indicated.

As the objective of the intervention was to identify immediate HRSNs, screening questions on the initial call were focused on anticipated common barriers to home isolation, including access to food, medication, and housing. To our knowledge, at the time of the study no standardized questions for assessing COVID-19 isolation-specific HRSNs existed. For this reason, the study investigators, including the ED patient navigators, developed HRSN screening questions based on our collective experience. Although the questions were designed with a yes/no response structure in mind, PAs and RAs were trained to allow patients to respond as they saw fit and to record a “yes” if a need was indicated at any point during the response. Notably, all patients were offered the opportunity to speak to a patient navigator who was experienced and trained in conducting personalized HRSN screening and referral, as well as in providing resources.

On Days 4 and 8, telephone check-ins were conducted by a team of RAs in the ED. Given staffing changes and challenges associated with the pandemic, RAs were able to conduct follow-up screening and were supported by PA and physician back-up. Using standardized questionnaires in REDCap (a secure, web-based software platform designed to support data capture for research studies and hosted at Mass General Brigham), patients were re-screened for potential clinical worsening and for HRSNs (Table 1). Patients who reported worsening symptoms or any high-risk clinical symptom to the RA were called within one hour by a PA in the ED to determine whether the patient’s condition warranted either a return ED visit or an urgent appointment with their primary care physician or at the Respiratory Illness Clinic, which consisted of outpatient medical offices repurposed during the pandemic to serve as urgent care clinics for patients with respiratory symptoms. Lastly, we screened discharged patient who underwent COVID-19 testing for the presence of household contacts. Those patients who had household contacts received counseling that reflected US Centers for Disease Control and Prevention guidance on household strategies to reduce the risk of transmission to others in the home.

All Day 1 calls made by ED PAs were documented in the patient’s EHR during implementation of the intervention. For Day 4 and 8 calls, RAs documented the telephone encounters, which were compliant with the Health Insurance Portability and Accountability Act. REDCap provided the following for our study: 1) an intuitive interface for validated data capture; 2) audit trails for tracking data manipulation and export procedures; 3) automated export procedures for seamless data downloads to common statistical packages; and 4) procedures for data integration and interoperability with external sources.^15^, ^16^ For patients with concern for worsening clinical symptoms or identified HRSNs, ED PAs and patient navigators, respectively, documented in the patient’s EHR.

An algorithm was built into the REDCap questionnaire to allow just-in-time instructions for the RAs based on the responses they obtained from the patients. The talking points were embedded into the algorithm so that the RAs could have structured conversations with the patients, based on identified needs. Automatic flags were created in the tool to highlight patients who screened positive for potential clinical worsening and for unmet HRSNs. The preferred language of the patient was shown on the REDCap algorithm. and for non-English speakers a prompt would appear to initiate a call with an interpreter prior to contacting the patient.

### Data Collection

Retrospective chart review was conducted on all patients after the intervention period concluded. We collected demographic information from the EHR, including patient age, race, gender, primary language, and insurance status. The RT-PCR results for SARS-CoV-2 were recorded for all patients. For patients who screened positive for HRSNs, the type of social need was categorized and recorded into four predetermined domains: food insecurity; housing insecurity; utilities-related need; and medication-related need. Patient data was recorded using REDCap. We conducted analyses using SAS version 9.4 (SAS Institute Inc, Cary, NC).

### Analysis

Descriptive statistics summarized demographic information, SARS-CoV-2 RT-PCR test results, prevalence of HRSNs, and the prevalence of worsening clinical symptom. Given the exploratory nature of this study, comparative analyses were not performed.

### Institutional Review Board

This study was deemed exempt by the Mass General Brigham Institutional Review Board (Boston, MA).

## RESULTS

The program was active at our institution from April 24–June 29, 2020. During this period, calls were made to 1,445 unique patients discharged from the ED with a pending COVID-19 test. Characteristics of our patient population are presented in Table 2. The average age of patients was 48.5 years. On Day 1, 1,468 calls to 1,445 unique patients were made (several patients had return visits and were called after each ED visit). Due to the evolving nature of the COVID-19 pandemic and the need to rapidly stand up the program the number of patients reached by the PAs on Day 1 was not recorded.

**Figure 1:**
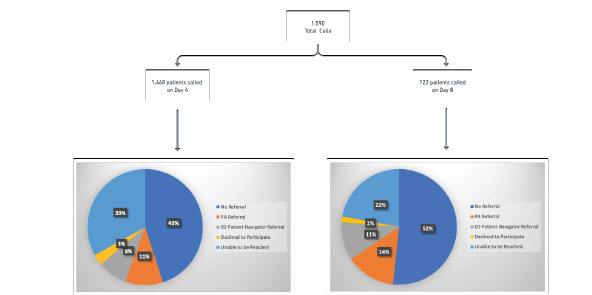
Outcome of day 4 and day 8 patient calls: April 24–June 29, 2020. Note: On Day 4, 1,468 calls to 1,445 unique patients were made (several patients had return visits and were called after each ED visit). Day 8 calls were started on May 10, 2020, and call attempts were made to 122 of the initial 1,445 patients. *PA*, physician assistant; *ED*, emergency department.

On Day 4, RAs reached 67.0% of patients called. Of these, 67.2% required no referral, 15.9% were referred to a PA out of concern for clinical worsening, 12.4% were referred to an ED patient navigator out of concern for HRSNs, and 4.5% of patients declined to participate. On Day 8, 81.8% of the 122 patients that were called were reached by the RAs. Of these, 62.8% required no referral, 19.7% were referred to a PA out of concern for clinical worsening, 14.0.% were referred to an ED patient navigator out of concern for HRSNs, and 2.3% declined to participate (Figure 1).

### Post-discharge Clinical Needs

Of the patients who were reached and willing to participate on Day 4 and Day 8 calls, 16.4% (173 patients) screened positive for worsening clinical status and required a telehealth check-in with the ED PAs. Of all the patients referred to a PA for clinical reassessment, 31.8% had tested positive for COVID-19 (Table 3). Of the patients who tested positive, 27.3% were White, 23.6% were Black or African American, 7.3% were Asian, and 41.8% were characterized as other race; 49% were Latinx, 45.5% were non-Latinx, and 5.5% of patients’ ethnicity was not available. Patients identifying as Black, other, or Latinx, were referred to ED patient navigators at disproportionately higher frequency compared to those who identified as White or Asian.

Of the patients subsequently reached by a PA for reassessment, only 14 (0.95% of total population) were referred back to the ED or to the Respiratory Illness Clinic. Of note, the total number of patients reached by the PAs on Day 1 or on Day 4 and 8 follow-up calls was not recorded; thus, we were unable to assess percentage of patients reached (Figure 1).

### Health-related Social Needs of Patients

We found that 12.6% (n = 135) of patients reached on Day 4 or Day 8 calls screened positive for HRSNs and were referred by the RA to an ED patient navigator. Of these 135 patients, 26.7% had tested positive for COVID-19, 56.3% were subsequently reached by a patient navigator, and 33.3% could not be reached. In 10.0% of patients, outreach was deferred because they were already being followed closely by their outpatient team for HRSNs (Table 4). Of the 76 patients reached by an ED patient navigator, 89.5% were identified as having HRSNs, 31.1% were Black or African American, 17.0% were White, and 50.4% were characterized as other race. Of patients referred to a patient navigator for HRSNs, 48.1% were Latinx and 48.1% were non-Latinx (Table 3).

**Figure 2:**
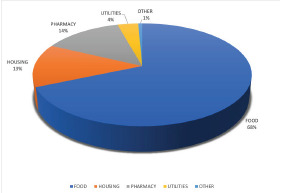
Categorization of social support referrals.

Among the 76 patients who were reached by an ED patient navigator, 110 referrals were placed to address HRSNs. Among these patients, 46.1% required referral from one HRSN domain, 35.5% required referrals from two HRSN domains, 3.9% required referrals from three HRSN domains, and 3.9% required referral from four HRSNs domains; 10.5% did not require referral. Of the HRSNs that were addressed by the patient navigators, the majority were related to food and housing insecurity, as well as difficulty obtaining medications (Table 5). Of the total referrals to address these needs, 68.6% were for food resources, 13.6% for housing resources, 13.6% for medication-related resources, and 3.6% for utilities resources (Figure 2); 72.3% of these referrals were to non-government programs, and 27.7% were to government programs.

## DISCUSSION

The purpose of this QI project was to screen and provide referral for HRSNs and conduct clinical screening for worsening symptoms. We found that nearly as many patients required referral for unmet social needs as for clinical reassessment. Despite the high number of referrals placed by RAs, the PAs did not identify many patients who required emergent, in-person evaluation. Most clinical needs were resolved through discussion over the phone. Based on feedback received from PAs, the difference in the number of patients referred for PA telephone screening and the number referred for in-person care likely reflects several factors, including patient uncertainty surrounding the diagnosis of COVID-19 at the start of the pandemic, lack of clarity about discharge/isolation instructions, and a high number of inquiries regarding non-COVID-19 related medical concerns. (The RAs were instructed to refer anyone with a medical concern to a PA to avoid mistriage.) Although few patients ultimately required in-person re-evaluation, we did informally observe that these PA follow-up calls helped to clarify discharge instructions and recognize challenges with adherence, which may have reduced re-presentation to the ED.

Notably, a high percentage of patients required referral for HRSNs that influenced their ability to safely isolate and quarantine. This mirrors statewide data for Massachusetts, where 17% of cases and contacts in the community tracing collaborative contact-tracing system were referred to social support to help them isolate.^14^ In other communities nationwide, the percentage of patients requiring support to safely isolate and quarantine has been reported to be as high as 72%.^17^ While identifying and addressing patient social support needs during contact tracing is an important component of a public health response, the earlier that social needs can be identified and addressed to allow safe isolation, the greater the impact will be.^18^,^19^ This program screened for social needs on Day 1 after discharge, but programs to incorporate similar screening at the time of the ED visit should be considered. Although not quantifiable, program staff also engaged in risk-reduction conversations and answered questions regarding self-isolation and quarantine, which likely further enhanced patients’ ability to safely isolate.

Our data shows that a greater proportion of patients who identified as Black or “other” race, as well as those identifying as Latinx ethnicity, were referred to ED patient navigators. Similarly, a disproportionately higher number of Latinx patients were referred to the ED PAs. This likely reflects underlying structural inequities, including wealth and housing, access to primary care, as well as the disproportionate impact of the COVID-19 illness burden on minoritized patient populations. Our findings are consistent with prior studies that have demonstrated high unmet HRSNs within these patient populations. 20

The greatest HRSN identified by our ED patient navigators was food insecurity, which has been associated with more frequent ED visits and worse health outcomes. 21 Since our questions focused on immediate needs for the duration of the quarantine and isolation period, the true burden of food insecurity is likely even higher than our results. Future programs should evaluate ways to identify and reduce food insecurity for ED patients. Importantly, screening programs need to engage individuals who can help patients navigate available resources, such as the ED patient navigators in our program. Further expansion of these programs, including beyond Medicaid ACOs, should be considered. In addition, the success of ED patient navigators can be facilitated through developed resource lists outlining existing community programs, eligibility requirements, and instructions on how to access resources. In our program, these lists were developed by our ED patient navigators, but they could also be produced at an institutional or municipal level.

## LIMITATIONS

Several limitations must be considered when interpreting the results. First, this was designed as a QI project during the first peak of the COVID-19 pandemic, rather than as a research study; therefore, there may have been non-controlled confounders. Due to an absence of validated, short HRSN screenings focused on COVID-19 isolation needs at the time of the project, we designed HRSN questions based on detailed knowledge of social determinants of health and immediate needs associated with safe COVID-19 isolation, which had not been validated. While our overall rates of missing data were low, there were gaps in data collection that reflected the retrospective nature of the study.

Demographic information was taken from the EHR system, which can sometimes be inaccurate. For example, patients may mistakenly report their ethnicity under race; hence, a large percentage of patients’ race was characterized as “other.” We do not have information on patients whom we were unable to reach or those who declined to participate; it is possible these patients could have been more or less likely to require referral. This is particularly relevant in the case of patients who were unable to be reached, as those without stable access to a phone would have been less likely to be reached but may have had greater social needs. As previously noted, due to the rapidly evolving pandemic and need to quickly stand up the program, the number of patients reached by PAs on Day 1 was not recorded; thus, we were unable to evaluate the referral outcomes for these calls, as was done with the calls done by RAs.

Finally, this project was run early in the COVID-19 pandemic, when guidance on testing, isolation, and quarantine was rapidly evolving. As a result, protocol variations were necessary throughout the program’s existence, such as the shift to calling only positive patients on June 10. Similarly, testing criteria and isolation/quarantine guidelines were also changing, and these variations may have affected the consistency and effectiveness of our intervention.

## CONCLUSION

In this patient population, the demand for interventions to address social needs was as high as the need for clinical reassessment. By leveraging existing systems, we were able to use patient navigators in the ED to perform health-related social needs assessments and address urgent needs. Development of an ED-based telehealth program to monitor symptom progression and unmet HRSNs is feasible; similar ED-based programs should be considered to help support patients’ interdependent social and health needs, beyond those related to COVID-19.

## Figures and Tables

**Table 1:** Screening questions for health-related social needs.

Do you have enough food, medications, and necessities for the next 7–14 days?
Do you have someone who can bring you food, medications, or household necessities if needed?
Will you be able to isolate safely in your own home for the next 10 days?
Would you like resources to help you with obtaining food, medications, household necessities, or housing?

**Table 2:** Demographics of patients discharged from the emergency department with pending COVID-19 test.

	Number of individual patients N = 1,445 (%)
Language	
English	1,191 (81.0%)
Spanish	191 (13.0%)
Haitian Creole	13 (0.9%)
Russian	8 (0.5%)
Other	75 (5.1%)
Gender	
Female	848 (57.7%)
Male	622 (42.3%)
Race	
White	647 (44.8%)
Black or African American	359 (24.8%)
Other	332 (23.0%)
N/A	59 (4.1%)
Asian	42 (2.9%)
American Indian or Alaska Native	6 (0.4%)
Ethnicity	
Non-Latinx	1,002 (69.3%)
Latinx	356 (24.6%)
N/A	87 (6.0%)
Insurance	
Private	502 (34.2%)
Medicaid	326 (22.2%)
Medicare	314 (21.4%)
Self-pay	11 (0.8%)
N/A	314 (21.4%)

**Table 3:** Referrals and COVID-19 status by race and ethnicity.

	Total Patients Referred to ED Patient Navigator (n =135) (%)	Total Patients Referred to PA (n = 173) (%)	All Patients Called (n = 1,445)
Race			
Asian	2 (1.5%)	10 (5.8%)	42 (2.9%)
Black	42 (31.1%)	39 (22.5%)	359 (24.8%)
White	23 (17.0%)	68 (39.3%)	647 (44.8%)
American Indian or Alaska Native	0 (0%)	0 (0%)	6 (0.4%)
Other	68 (50.4%)	56 (32.4%)	332 (23.0%)
Ethnicity			
Latinx	65 (48.15%)	59 (34.1%)	356 (24.6%)
Non-Latinx	65 (48.15%)	108 (62.4%)	1,002 (69.3%)
N/A	5 (3.7%)	6 (3.5%)	87 (6.0%)
COVID-19 Status			
Positive	36 (26.7%)	55 (31.8%)	215 (15%)
Negative	99 (73.3%)	118 (68.2%)	1253 (85%)

*ED*, emergency department; *PA*, physician assistant; *COVID-19*, coronavirus disease 2019.

**Table 4:** Outcome of telephone calls made by patient navigators in the emergency department.

	Number of patients (N = 135) (%)
Patients reached by ED patient navigator	76 (56.3%)
Patients unable to be reached	45 (33.3%)
Patients with HRSN already being addressed per chart review (call deferred)	14 (10.0%)

*HRSN*, health-related social needs.

**Table 5:** Type of health-related social needs addressed by patient navigators in the emergency department.

	Number of times HRSN was addressed (N = 110) (%)
Food	51 (46.6%)
Housing	17 (15.4%)
Medication Delivery	14 (12.7%)
Paying for Medications	8 (7.2%)
Legal Assistance	5 (4.5%)
Paying Utility Bills	3 (2.7%)
Job Search or Training	2 (1.8%)
Care for Elder or Disabled	1 (0.9%)
Violence Prevention	1 (0.9%)
Childcare	0 (0.0%)
Transportation	0 (0.0%)
Other	8 (7.2%)

*ED*, emergency department; *HRSN*, health-related social needs.
